# Development of a nomogram for predicting recurrence of epithelial ovarian cancer involving traditional Chinese medicine treatment

**DOI:** 10.3389/fonc.2025.1577110

**Published:** 2025-05-08

**Authors:** Xiaofeng Chen, Xudong Hu, Huanmei Lin, Ziang Li, Baijun Gao, Hongmei Ouyang, Xiangdan Hu, Jing Xiao

**Affiliations:** ^1^ The Second Clinical College of Guangzhou University of Chinese Medicine, Guangzhou, Guangdong, China; ^2^ Department of Gynecology, The Second Affiliated Hospital of Guangzhou University of Chinese Medicine, Guangzhou, Guangdong, China; ^3^ Yuexiu District Maternal and Child Health Hospital, Guangzhou, Guangdong, China; ^4^ State Key Laboratory of Traditional Chinese Medicine Syndrome/Department of Gynecology, The Second Affiliated Hospital of Guangzhou University of Chinese Medicine, Guangzhou, Guangdong, China

**Keywords:** ovarian cancer, nomogram, predictive model, recurrence, traditional Chinese medicine

## Abstract

**Background:**

The treatment of epithelial ovarian cancer (EOC) is evolving towards personalization and precision. Early prediction of recurrence can provide a basis for individualized monitoring and treatment. Our study aims to develop a predictive model for early recurrence of ovarian cancer incorporating Traditional Chinese Medicine (TCM) treatment.

**Methods:**

We reviewed the clinicopathological and prognostic data of EOC patients who achieved complete clinical remission after surgery and chemotherapy at Guangdong Traditional Chinese Medicine Hospital (GPHCM) between December 2011 and July 2022. Basic information, clinical characteristics, treatment plans, and follow-up data were collected. Univariate logistic analysis was performed to identify significant variables (*P*<0.10), followed by Least Absolute Shrinkage and Selection Operator (LASSO) regression to further determine key risk factors. A multivariate logistic regression model was constructed based on these factors, and a nomogram was developed to predict recurrence risk. The model’s effectiveness was internally validated using bootstrap resampling (1000 iterations) and assessed for discrimination and calibration using Area Under Curve (AUC), the Hosmer-Lemeshow test, and calibration plots. Additionally, decision curve analysis (DCA) was performed to evaluate the clinical utility of the model.

**Result:**

This study included a total of 170 patients. Multivariate logistic regression analysis revealed that surgical procedure, The International Federation of Gynecology and Obstetrics (FIGO) stage, completion of the full chemotherapy course, and exposure to TCM were independent prognostic factors for ovarian cancer recurrence. Based on these factors, this study developed a nomogram model to predict recurrence risk, incorporating four key variables. The AUC of the prediction model was 0.843 (95% CI: 0.774-0.898), and the Hosmer-Lemeshow test and calibration plot indicated good calibration. DCA showed the model provided higher net benefit across a wide range of threshold probabilities.

**Conclusion:**

The nomogram we developed effectively predicted 2-year recurrence risk in epithelial ovarian cancer patients. Notably, TCM treatment lasting more than 6 months may help prolong progression-free survival (PFS).

## 1 Introduction

Ovarian cancer is a primary malignant tumor originating from the ovaries or fallopian tubes, accounting for approximately 6.7% of new cancer diagnoses and 4.0% of cancer-related deaths worldwide in 2022 (1). Epithelial ovarian cancer (EOC) is the most common histological type of ovarian cancer, accounting for approximately 80% of ovarian malignancies (2). Compared to other ovarian cancer subtypes, it has a higher incidence and mortality rate (3). Currently, the standard treatment for patients with newly diagnosed advanced EOC involves cytoreductive surgery followed by platinum-based combination chemotherapy (4). Although 59% to 81% of patients respond to first-line chemotherapy, approximately 75% of patients will experience recurrence within a median time of 18 to 24 months in the absence of maintenance therapy (5). After recurrence, ovarian cancer exhibits increasing therapeutic resistance, leading to a significant reduction in progression-free survival (PFS) and poor overall prognosis (OS). Therefore, early prediction and assessment of recurrence in EOC can provide healthcare professionals with a basis for individualized monitoring and proactive treatment strategies, which may help extend PFS.

In China, Traditional Chinese Medicine (TCM) has been widely used and recognized in cancer treatment. TCM therapies, including acupuncture, herbal medicine, and tui na (therapeutic massage), have played an important role in the prevention and treatment of ovarian cancer (6, 7). Existing studies have shown that TCM can not only alleviate the side effects of radiotherapy and chemotherapy (8–11), but also prolong patients’ survival (12). Chinese ovarian cancer diagnosis and treatment guidelines have incorporated the process of integrating TCM into ovarian cancer treatment (13). However, its global adoption remains limited due to factors such as insufficient high-quality clinical evidence, regulatory differences, and varying levels of acceptance across different healthcare systems. Based on this, this study innovatively included TCM exposure as a candidate variable in constructing the recurrence prediction model, aiming to provide evidence-based support for the role of TCM in preventing ovarian cancer recurrence.

The nomogram, as a prognostic prediction tool, integrates multiple clinical variables to provide individualized prognostic assessment in the form of a quantitative score. Compared to traditional staging systems, the nomogram offers more intuitive and personalized predictions (14). Although several ovarian cancer recurrence prediction models have been developed (15, 16), most are based on public databases with limited data and insufficient clinical details, making it difficult to achieve precise individualized predictions. Therefore, this study utilized clinical data from patients at our center and applied logistic regression along with the least absolute shrinkage and selection operator (LASSO) machine learning algorithm (17) to construct an individualized nomogram model for predicting the 2-year recurrence risk in EOC patients. The development of this model not only provides clinicians with more valuable decision-making tools but also holds potential for application in optimizing treatment plans and personalized prognostic assessments.

## 2 Methods

**Figure 1:**
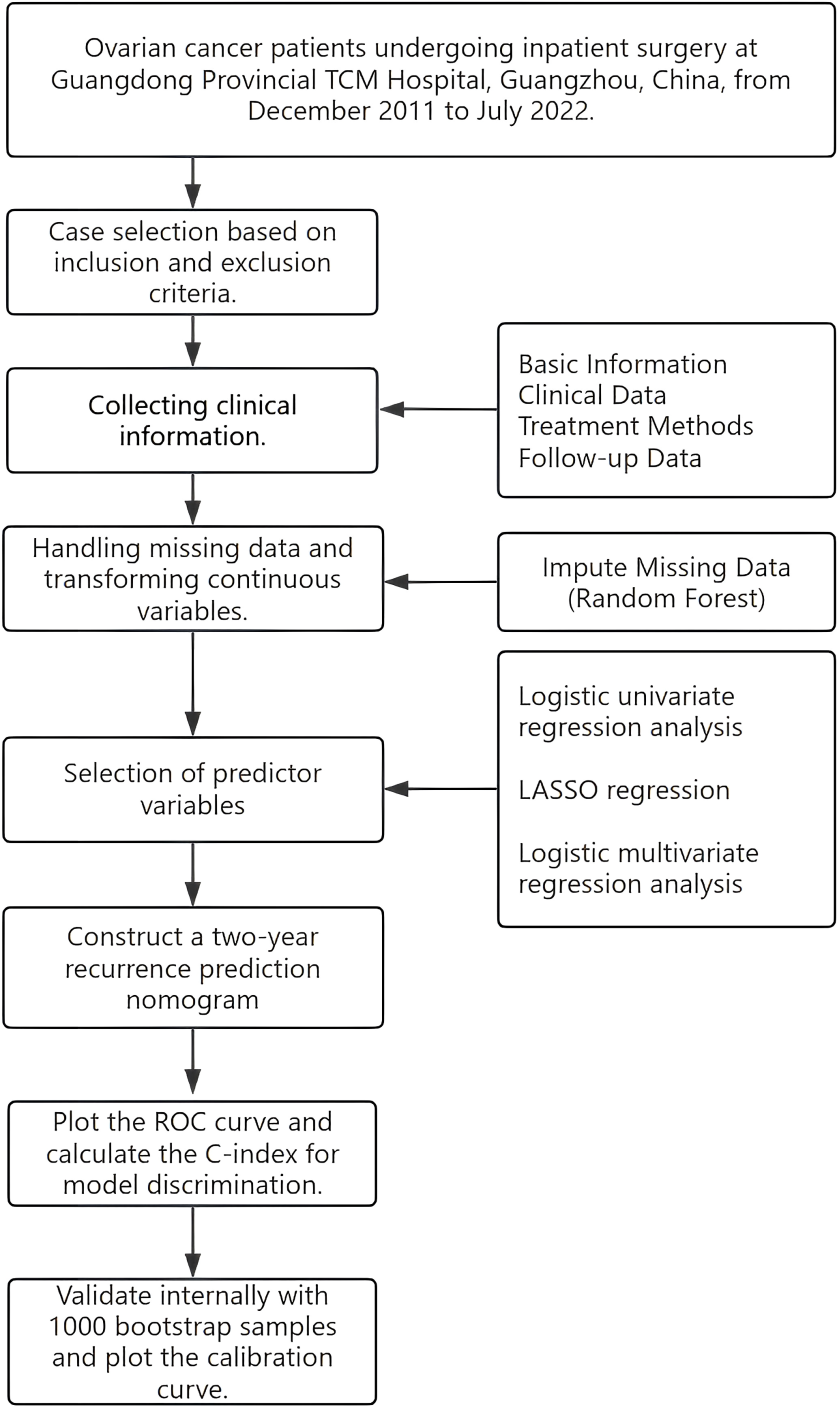
Flowchart of the study design.

This study conducted a retrospective analysis of EOC patients who were hospitalized and treated at the four branches of Guangdong Traditional Chinese Medicine Hospital (GPHCM) from December 2011 to July 2022, with follow-up extending until November 2024. The study design flowchart is shown (**Figure 1**). The ethics committee of GPHCM reviewed and approved the study protocol (ethics number:YE2022-116-01). Given the retrospective nature of this study, patient identity information was anonymized, and informed consent was not required.

### 2.1 Inclusion and exclusion criteria

#### 2.1.1 Inclusion criteria

1. Patients diagnosed with EOC by histopathological examination.
2. No history of other malignancies.
3. Patients had no prior cancer treatment at other institutions before receiving surgery or neoadjuvant chemotherapy at our institution.
4. Patients undergoing their first ovarian cancer surgery.
5. Age ≥ 18 years.

#### 2.1.2 Exclusion criteria

1. Patients who underwent fertility-sparing surgery.
2. Patients with significant missing clinical data.
3. Patients with incomplete follow-up, where recurrence outcomes could not be determined.

### 2.2 Data collection and definition

#### 2.2.1 General information

Data collection was carried out by two researchers independently, who reviewed and recorded the data separately. Discrepancies between them were resolved by a third researcher. The collected clinical information included: age, height, weight, menopausal status, comorbidities, cancer family history, pre-treatment CA125 levels, receipt of neoadjuvant chemotherapy, surgical approach, surgical procedure, surgical duration, blood loss during surgery, intraoperative chemotherapy, use of intraperitoneal and intravenous chemotherapy (IP & IV chemo), use of intraperitoneal hyperthermic (HIPEC) perfusion, ovarian tumor laterality, histopathological type, International Federation of Gynecology and Obstetrics (FIGO) stage, lymph node involvement, postoperative chemotherapy regimen, chemotherapy regimen, chemotherapy dose adjustments, completion of all chemotherapy cycles, chemotherapy adherence, whether BRCA/HRD genetic testing was performed, and use of targeted therapy.

#### 2.2.2 Definition and classification of TCM intervention exposure

The outpatient and inpatient medical record system at GPHCM provides detailed documentation of each patient’s pharmacological treatments and TCM prescriptions throughout their hospital stay and clinical visits, including information on drug names, dosages, initiation dates, frequency, duration, and routes of administration. In this study, the Chinese herbal decoctions prescribed to the patients were limited to those prescribed by senior TCM practitioners from the gynecology or oncology departments based on the patient’s syndrome differentiation. Chinese herbal decoctions, a fundamental principle of TCM, are prescribed by TCM doctors based on each patient’s symptoms and tongue-pulse diagnosis, with tailored herbal formulas aimed at treating the patient’s specific condition and presenting symptoms (18). In the present study, TCM therapy predominantly included a range of commonly used Chinese herbal medicines, such as Ren Shen (Panax ginseng), Chai Hu (Radix Bupleuri), Bai Zhu (Atractylodes macrocephala), Fu Ling (Poria cocos), and Ban Xia (Pinellia ternata). Classical herbal formulas frequently used in clinical practice, including Xiao Chai Hu Tang (Minor Bupleurum Decoction), Xue Fu Zhu Yu Tang (Drive Out Stasis in the Mansion of Blood Decoction), and Liu Jun Zi Tang (Six Gentlemen Decoction), were commonly prescribed and adjusted based on syndrome differentiation.

Data entry was performed independently by two researchers, who conducted cross-checking of the data. The researchers were required to calculate the cumulative duration of TCM herbal decoction use for each patient from the diagnosis of ovarian cancer to the occurrence of the study outcome. Based on the classification of TCM exposure in previous literature (18, 19), high exposure was defined as a cumulative use of TCM herbal decoctions for at least 6 months, while less than 6 months was considered no or low exposure.

#### 2.2.3 Follow-up and outcome assessment

The researchers conducted follow-up through telephone consultations, outpatient visits, and inpatient medical record inquiries. The follow-up schedule was as follows: every 3 months during the first 1 to 2 years after treatment; every 3 to 6 months for the next 3 years; and annually after 5 years. The follow-up included assessment of symptoms (such as bloating, loss of appetite, weight loss, abnormal bowel movements, or abdominal masses), signs (such as pleural and peritoneal effusion, gynecological examination revealing masses, or bowel obstruction), tumor markers including serum CA125 and serum HE4 levels, complete blood count, imaging studies of the abdomen and pelvis, and transvaginal gynecological ultrasound. If necessary, abdominal Magnetic Resonance Imaging (MRI), pelvic MRI, or Positron emission tomography/computedtomography (PET-CT) were performed. Recurrence was defined as CA125 levels above the normal value (35 U/mL) with persistent elevation or metastasis detected on imaging studies. The follow-up period ended in November 2024. The primary endpoint was PFS, defined as the time from the start of treatment to tumor progression or patient death. The main study endpoint was the 2-year PFS rate.

### 2.3 Statistical analysis

Statistical analysis was performed using R software version 4.4.2 (20). The missing data rate in this study was 0.5%, with the missing rate for each variable being less than 20%. Missing data were imputed using the random forest imputation method from the ‘missForest’ package in R. Categorical data were described as percentages (%) or proportions. Intergroup comparisons were conducted using chi-square tests or Fisher’s exact tests (depending on sample size and expected frequencies). Normally distributed continuous data were presented as mean ± standard deviation (SD), and comparisons between groups were made using independent samples t-tests. Non-normally distributed continuous data were presented as median (Q1, Q3), and intergroup comparisons were performed using the Mann-Whitney U test. All variables were considered potential predictors. Univariate logistic regression was used to identify significant predictors (*P*<0.1), which were then included in a LASSO regression model to eliminate multicollinearity and simplify the model. Cross-validation was used to select the optimal regularization parameter λ. A multivariate logistic regression model was used to develop a nomogram for predicting the 2-year recurrence risk of EOC. The model’s predictive performance was assessed by calculating the area under the curve (AUC) of the receiver operating characteristic (ROC) curve. Internal validation was performed using bootstrap resampling with 1000 iterations, and the 95% confidence intervals were calculated for the model’s performance. The model’s accuracy was evaluated using the Hosmer-Lemeshow goodness-of-fit test, and the results were visualized by plotting calibration curves. To assess the robustness of the model, sensitivity analyses were conducted in two distinct datasets: the complete-case dataset (excluding all records with missing data), and a separate dataset consisting of patients who had undergone breast cancer susceptibility gene (BRCA) or homologous recombination deficiency (HRD) genetic testing. Decision curve analysis (DCA) (21) was used to assess the clinical utility of the model.

## 3 Results

Based on the inclusion and exclusion criteria, a total of 170 patients were included in this study, of which 100 (58.82%) had no recurrence and 70 (41.18%) experienced recurrence. The general characteristics and clinical features of the non-recurrence group and recurrence group are shown in **Table 1**.

**Table 1:** Demographic and clinical characteristic of study participants.

Characteristic	Total (n = 170)	Non-relapse Group (n = 100)	Relapse Group (n = 70)	*P*
Age, Mean ± SD	51.22 ± 10.1	50.17 ± 11.2	52.71 ± 8.12	0.088
Height, Mean ± SD	156.71 ± 5.85	156.7 ± 6.11	156.73 ± 5.49	0.970
Weight, Mean ± SD	51.99 ± 7.26	52.1 ± 7.16	51.82 ± 7.44	0.805
Menopausal, n (%)				0.068
No	81 (48)	54 (54)	27 (39)	
Yes	89 (52)	46 (46)	43 (61)	
Comorbidities, n (%)				0.261
Diabetes mellitus	4 (2)	3 (3)	1 (1)	
Hypertension	17 (10)	12 (12)	5 (7)	
No comorbidities	142 (84)	79 (79)	63 (90)	
Thyroid disease	7 (4)	6 (6)	1 (1)	
Cancer family history, n (%)				0.451
No	137 (81)	83 (83)	54 (77)	
Yes	33 (19)	17 (17)	16 (23)	
CA125, Median (Q1,Q3)	235.35 (64.32, 901.2)	114.95 (44.8, 571.05)	603.05 (102.83, 1600.25)	< 0.001*
Neoadjuvant chemotherapy (NACT), n (%)				< 0.001*
No	133 (78)	90 (90)	43 (61)	
Yes	37 (22)	10 (10)	27 (39)	
Surgical Approach, n (%)				0.011*
Laparoscopic surgery	28 (16)	23 (23)	5 (7)	
Open abdominal surgery	142 (84)	77 (77)	65 (93)	
Surgical procedure, n (%)				< 0.001*
Comprehensive staging surgery	104 (61)	80 (80)	24 (34)	
Initial cytoreductive surgery	29 (17)	11 (11)	18 (26)	
Interval debulking surgery	37 (22)	9 (9)	28 (40)	
Surgical duration, Mean ± SD	311.51 ± 81.58	314.21 ± 80.98	307.64 ± 82.86	0.608
Blood loss, Median (Q1,Q3)	200 (100, 500)	200 (100, 300)	400 (200, 600)	< 0.001*
Intraop chemotherapy, n (%)				0.231
No	164 (96)	98 (98)	66 (94)	
Yes	6 (4)	2 (2)	4 (6)	
IP&IV chemo, n (%)				1.000
No	159 (94)	93 (93)	66 (94)	
Yes	11 (6)	7 (7)	4 (6)	
Postop HIPEC, n (%)				0.107
No	133 (78)	83 (83)	50 (71)	
Yes	37 (22)	17 (17)	20 (29)	
Tumor laterality, n (%)				0.009*
Bilateral	51 (30)	21 (21)	30 (43)	
Left sided	60 (35)	39 (39)	21 (30)	
Right sided	59 (35)	40 (40)	19 (27)	
Histologic type, n (%)				< 0.001*
clear cell	30 (18)	22 (22)	8 (11)	
Endometrioid	22 (13)	20 (20)	2 (3)	
Mixed Type	9 (5)	7 (7)	2 (3)	
Mucinous	8 (5)	7 (7)	1 (1)	
Serous	101 (59)	44 (44)	57 (81)	
FIGO stage, n (%)				< 0.001*
1	62 (36)	57 (57)	5 (7)	
2	22 (13)	11 (11)	11 (16)	
3	70 (41)	25 (25)	45 (64)	
4	16 (9)	7 (7)	9 (13)	
Lymph node involvement, n (%)				< 0.001*
None	97 (57)	76 (76)	21 (30)	
Not Performed	35 (21)	13 (13)	22 (31)	
Present	38 (22)	11 (11)	27 (39)	
Postop chemotherapy, n (%)				0.270
0	1 (1)	0 (0)	1 (1)	
2	3 (2)	0 (0)	3 (4)	
3	13 (8)	9 (9)	4 (6)	
4	38 (22)	25 (25)	13 (19)	
5	15 (9)	7 (7)	8 (11)	
6	86 (51)	52 (52)	34 (49)	
7	12 (7)	6 (6)	6 (9)	
8	2 (1)	1 (1)	1 (1)	
Chemo regimen, n (%)				0.068
Other	8 (5)	3 (3)	5 (7)	
TC	154 (91)	95 (95)	59 (84)	
TP	8 (5)	2 (2)	6 (9)	
Chemo dose adjustment, n (%)				0.310
No	161 (95)	93 (93)	68 (97)	
Yes	9 (5)	7 (7)	2 (3)	
Chemo cycles completed, n (%)				< 0.001*
No	31 (18)	7 (7)	24 (34)	
Yes	139 (82)	93 (93)	46 (66)	
Chemo adherence, n (%)				0.469
No	146 (86)	88 (88)	58 (83)	
Yes	24 (14)	12 (12)	12 (17)	
BRCA/HRD tested, n (%)				0.361
No	139 (82)	79 (79)	60 (86)	
Yes	31 (18)	21 (21)	10 (14)	
Targeted drug, n (%)				0.602
No	144 (85)	83 (83)	61 (87)	
Yes	26 (15)	17 (17)	9 (13)	
TCM exposure, n (%)				0.060
High	64 (38)	44 (44)	20 (29)	
Low	106 (62)	56 (56)	50 (71)	

*p.value≤0.05.

### 3.1 Univariate ROC curve

**Figure 2:**
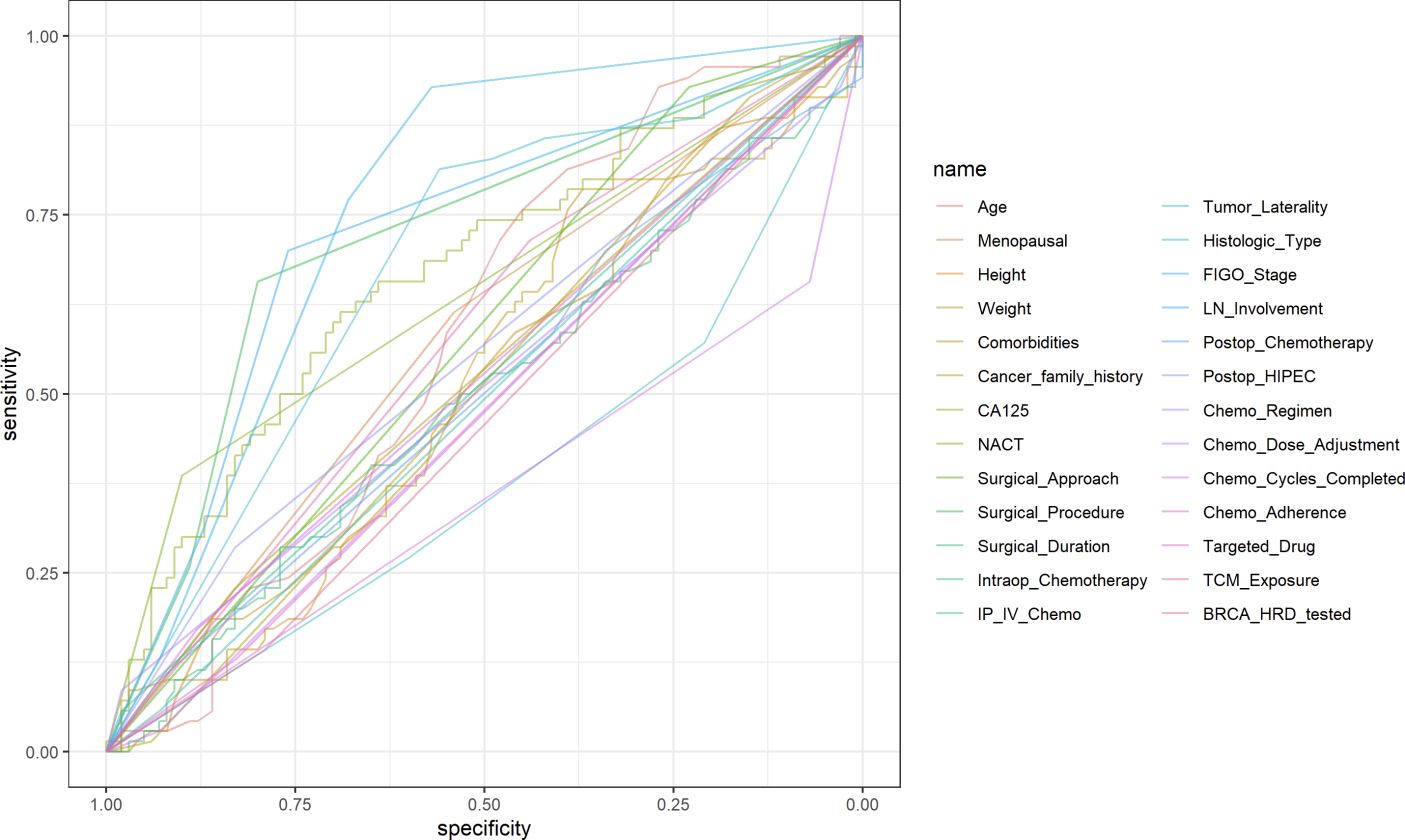
Univariate ROC curve for all potential variables.

**Figure 3:**
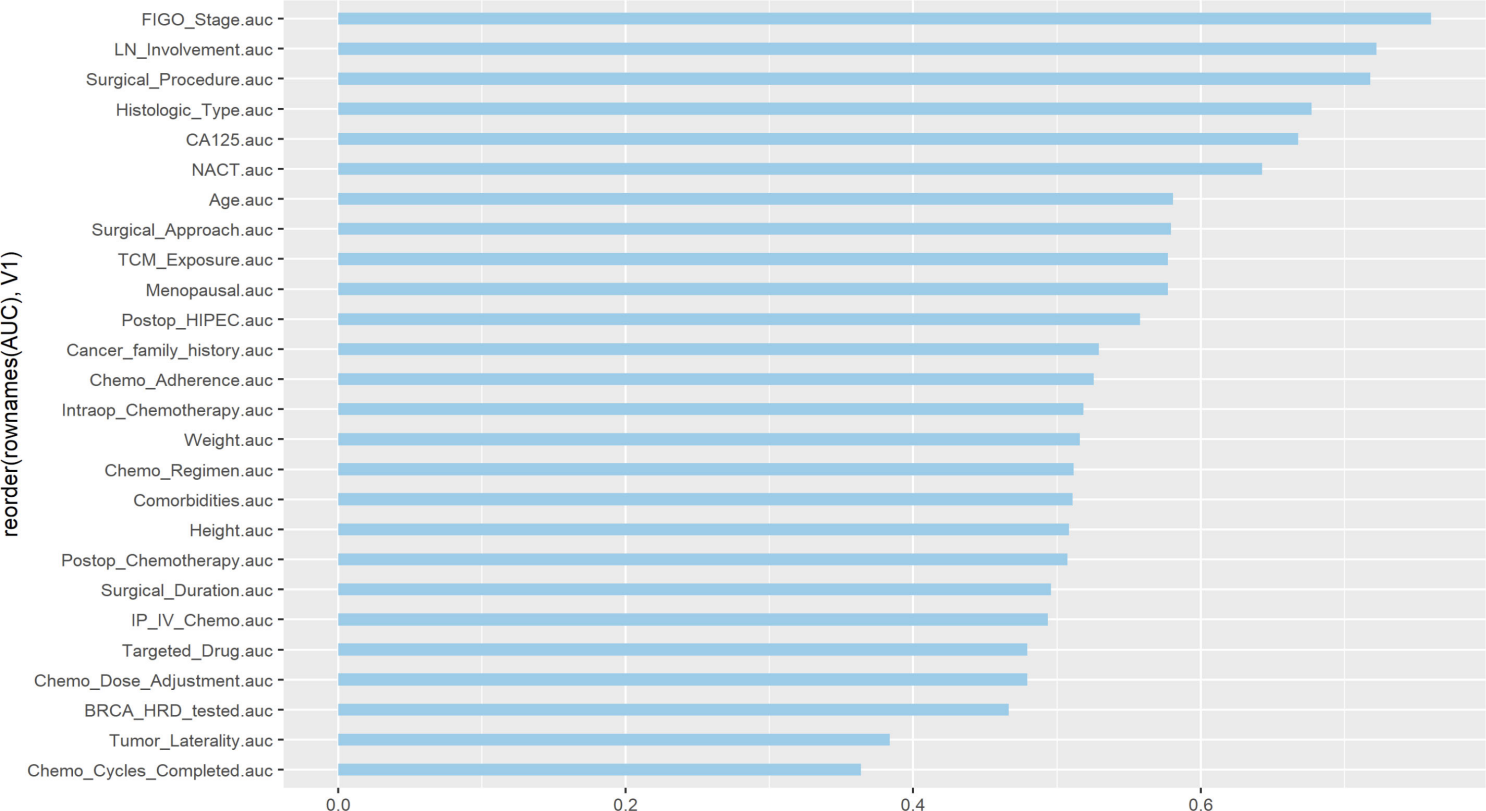
Visualization of univariate AUC values.

In this study, univariate ROC curves were plotted for all potential predictors (**Figure 2**), and their AUC results were visualized (**Figure 3**). The results showed that, except for FIGO stage (AUC = 0.7601429), none of the individual variables demonstrated significant predictive value for recurrence within 2 years in patients with EOC (0.25 < AUC < 0.75).

### 3.2 Selection of model variables

**Figure 4:**
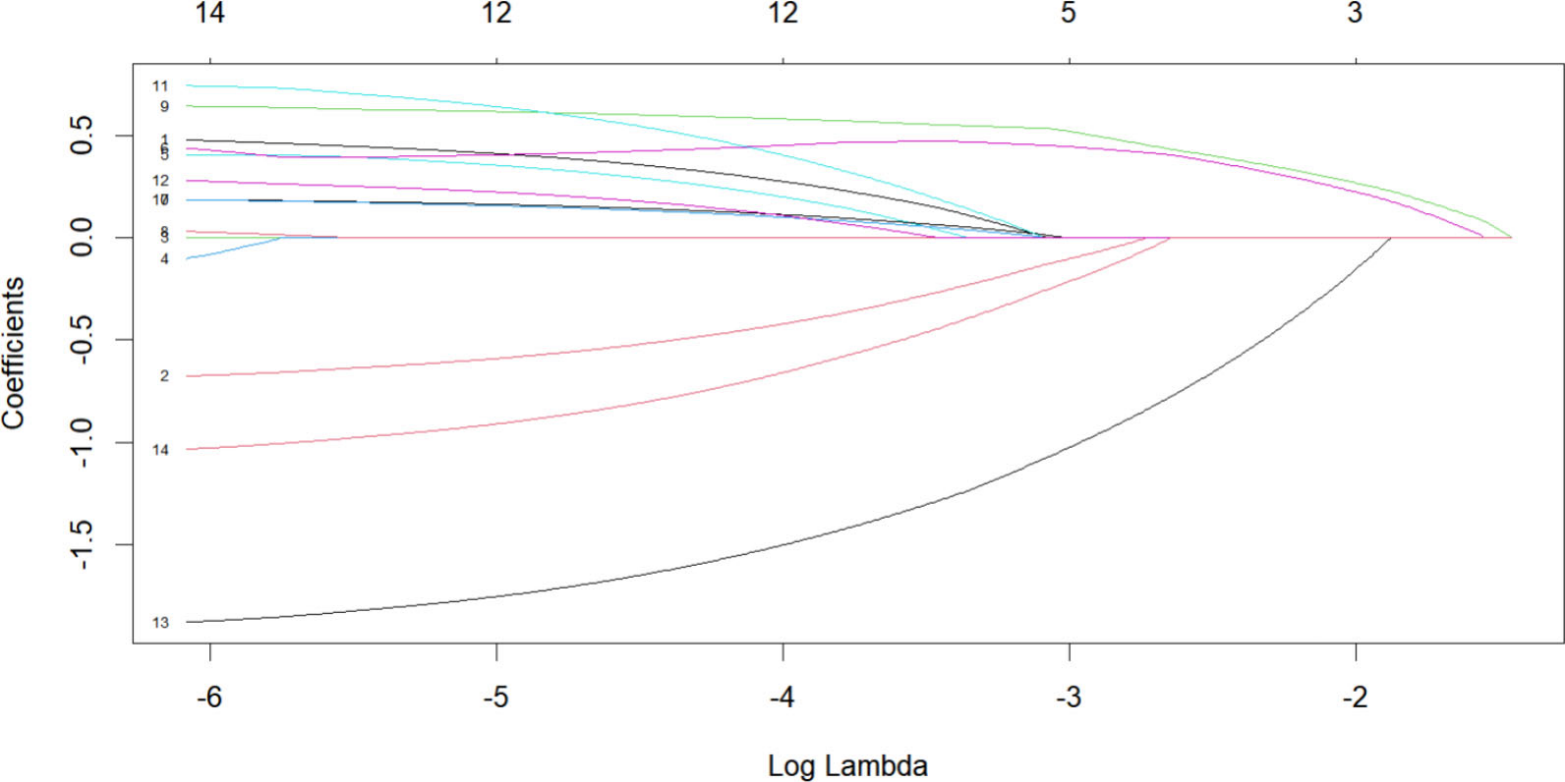
LASSO Coefficient distribution map-LASSO coefficient distribution of all variables.

**Figure 5:**
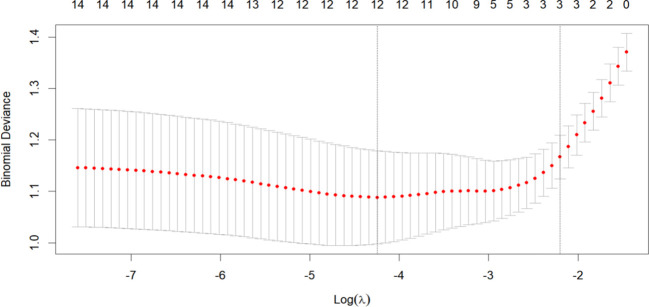
Variables determined by LASSO analysis (n=3).

To establish a 2-year recurrence prediction model for EOC, univariate logistic regression was performed for all potential predictors, as shown in **Table 2**. Based on the results of univariate logistic regression, further LASSO regression was conducted for variables with *P* < 0.1. Cross-validation was performed using the minimum criteria, and when the penalty coefficient λ = 0.01430764, the model demonstrated optimal performance with the fewest influencing factors. The selected variables (non-zero coefficients) included surgical procedure, FIGO stage, and completion of chemotherapy (**Figures 4**, **5**). Additionally, multivariate logistic regression was performed incorporating TCM exposure based on the three variables selected by LASSO. The multivariate logistic regression analysis showed that patients who underwent interval debulking surgery (OR = 1.827, 95% CI [0.58-5.76], *P*=0.07), were at a higher FIGO stage (OR = 2.429, 95% CI [1.42-4.15], *P*= 0.001), had incomplete chemotherapy (OR = 0.138, 95% CI [0.05-0.41], *P* < 0.001), or used TCM for less than 6 months (OR = 2.790, 95% CI [1.20-6.52], *P* = 0.018) had a higher recurrence rate (**Table 3**).

**Table 2:** Univariate logistic regression analysis of ovarian cancer recurrence risk.

Variables	β	S.E.	Z	OR	*P*.value
Age	0.026	0.016	1.608	1.026 ( 0.99 - 1.06 )	0.108
Height	0.001	0.027	0.037	1.001 ( 0.95 - 1.06 )	0.971
Weight	-0.005	0.022	-0.25	0.995 ( 0.95 - 1.04 )	0.803
Menopausal					
No	-0.626	0.317	-1.973	0.535 ( 0.29 - 0.99 )	0.048*
Yes	0.626	0.317	1.973	1.87 ( 1.01 - 3.51 )	0.048*
Comorbidities					
Diabetes Mellitus`	-0.758	1.165	-0.65	0.469 ( 0.02 - 3.75 )	0.515
Hypertension	-0.573	0.557	-1.028	0.564 ( 0.17 - 1.6 )	0.304
No Comorbidities`	0.872	0.468	1.864	2.392 ( 1 - 6.4 )	0.062*
Thyroid Disease`	-1.483	1.092	-1.358	0.227 ( 0.01 - 1.37 )	0.174
Cancer family history					
No	-0.369	0.39	-0.947	0.691 ( 0.32 - 1.49 )	0.343
Yes	0.369	0.39	0.947	1.447 ( 0.67 - 3.12 )	0.343
**CA125**	0	0	2.123	1 ( 1 - 1 )	0.034*
Neoadjuvant chemotherapy(NACT)					
No	-1.732	0.414	-4.183	0.177 ( 0.08 - 0.39 )	<0.001*
Yes	1.732	0.414	4.183	5.651 ( 2.58 - 13.25 )	<0.001*
Surgical approach					
Laparoscopic Surgery	-1.357	0.521	-2.602	0.258 ( 0.08 - 0.67 )	0.009*
Open Abdominal Surgery	1.357	0.521	2.602	3.883 ( 1.5 - 12.07 )	0.009*
Surgical procedure					
Comprehensive staging surgery	-2.037	0.355	-5.74	0.13 ( 0.06 - 0.26 )	<0.001*
Initial Cytoreductive Surgery`	1.03	0.421	2.448	2.801 ( 1.24 - 6.56 )	0.014*
Interval debulking surgery`	1.908	0.426	4.477	6.741 ( 3.02 - 16.32 )	<0.001*
**Surgical duration**	-0.001	0.002	-0.518	0.999 ( 1 - 1 )	0.605
IP&IV chemo					
No	0.217	0.647	0.335	1.242 ( 0.36 - 4.9 )	0.738
Yes	-0.217	0.647	-0.335	0.805 ( 0.2 - 2.78 )	0.738
Tumor laterality					
Bilateral	1.037	0.344	3.012	2.821 ( 1.45 - 5.6 )	0.003*
Left sided	-0.4	0.332	-1.206	0.67 ( 0.35 - 1.28 )	0.228
Right sided	-0.582	0.337	-1.724	0.559 ( 0.28 - 1.07 )	0.085*
Histologic type					
clear cell`	-0.782	0.447	-1.751	0.457 ( 0.18 - 1.06 )	0.08*
Endometrioid	-2.14	0.76	-2.817	0.118 ( 0.02 - 0.42 )	0.005*
Mixed Type	-0.94	0.818	-1.149	0.391 ( 0.06 - 1.68 )	0.25
Mucinous	-1.647	1.081	-1.524	0.193 ( 0.01 - 1.12 )	0.127
Serous	1.719	0.367	4.678	5.58 ( 2.78 - 11.84 )	<0.001*
**FIGO stage**	1.072	0.189	5.679	2.92 ( 2.05 - 4.32 )	<0.001*
Lymph node involvement					
None	-2	0.351	-5.706	0.135 ( 0.07 - 0.26 )	<0.001*
Not Performed`	1.121	0.393	2.85	3.067 ( 1.44 - 6.78 )	0.004*
Present	1.625	0.403	4.033	5.08 ( 2.36 - 11.59 )	<0.001*
**Postop chemotherapy**	-0.024	0.12	-0.198	0.977 ( 0.77 - 1.24 )	0.843
Postop HIPEC					
No	-0.669	0.375	-1.783	0.512 ( 0.24 - 1.07 )	0.075*
Yes	0.669	0.375	1.783	1.953 ( 0.94 - 4.11 )	0.075*
Other	0.911	0.748	1.219	2.487 ( 0.59 - 12.46 )	0.223
Chemo regimen					
TC	-1.265	0.564	-2.242	0.282 ( 0.09 - 0.82 )	0.025*
TP	1.525	0.832	1.832	4.594 ( 1.02 - 32.02 )	0.067*
Chemo dose adjustment					
No	0.94	0.818	1.149	2.559 ( 0.6 - 17.54 )	0.250
Yes	-0.94	0.818	-1.149	0.391 ( 0.06 - 1.68 )	0.250
Chemo cycles completed					
No	1.936	0.466	4.156	6.932 ( 2.91 - 18.51 )	<0.001*
Yes	-1.936	0.466	-4.156	0.144 ( 0.05 - 0.34 )	<0.001*
Chemo adherence					
No	-0.417	0.442	-0.943	0.659 ( 0.27 - 1.58 )	0.345
Yes	0.417	0.442	0.943	1.517 ( 0.63 - 3.64 )	0.345
BRCA/HRD tested					
No	0.467	0.421	1.11	1.595 ( 0.71 - 3.77 )	0.267
Yes	-0.467	0.421	-1.11	0.627 ( 0.27 - 1.40 )	0.267
Targeted drug					
No	0.328	0.445	0.736	1.388 ( 0.59 - 3.45 )	0.461
Yes	-0.328	0.445	-0.736	0.72 ( 0.29 - 1.69 )	0.461
TCM exposure					
High	-0.675	0.333	-2.03	0.509 ( 0.26 - 0.97 )	0.042*
Low	0.675	0.333	2.03	1.964 ( 1.03 - 3.82 )	0.042*

*: p.value≤0.100.

**Table 3:** Multivariate logistic regression analysis of ovarian cancer recurrence risk.

Variables	β	S.E.	Z	OR	*P*.value
Surgical procedure					
Comprehensive staging surgery	0			1 (Reference)	
Initial Cytoreductive Surgery`	0.6027	0.5856	1.029	1.827 (0.58 - 5.76)	0.303
Interval debulking surgery`	1.0536	0.5823	1.809	2.868 (0.92 - 8.98)	0.07
FIGO stage	0.8875	0.2732	3.248	2.429 (1.42 - 4.15)	0.001*
Chemo cycles completed					
No	0			1 (Reference)	
Yes	-1.9784	0.5481	-3.61	0.138 (0.05 - 0.41)	<0.001*
TCM exposure					
High	0			1 (Reference)	
Low	1.0262	0.4326	2.372	2.790 (1.20 - 6.52)	0.018*

*: p.value≤0.05.

### 3.3 Construction and validation of the nomogram prediction model

**Figure 6:**
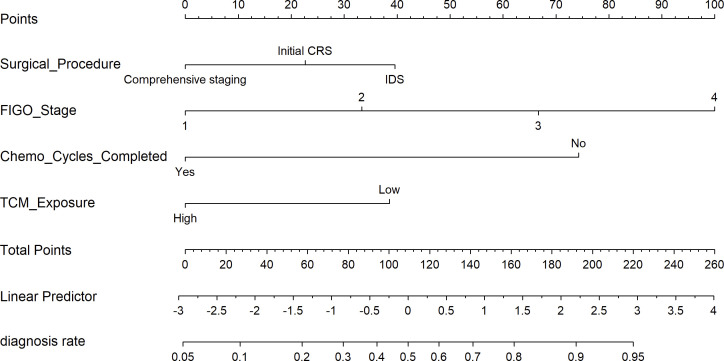
Nomogram for predicting 2-year recurrence in EOC patients after initial treatment.

**Figure 7:**
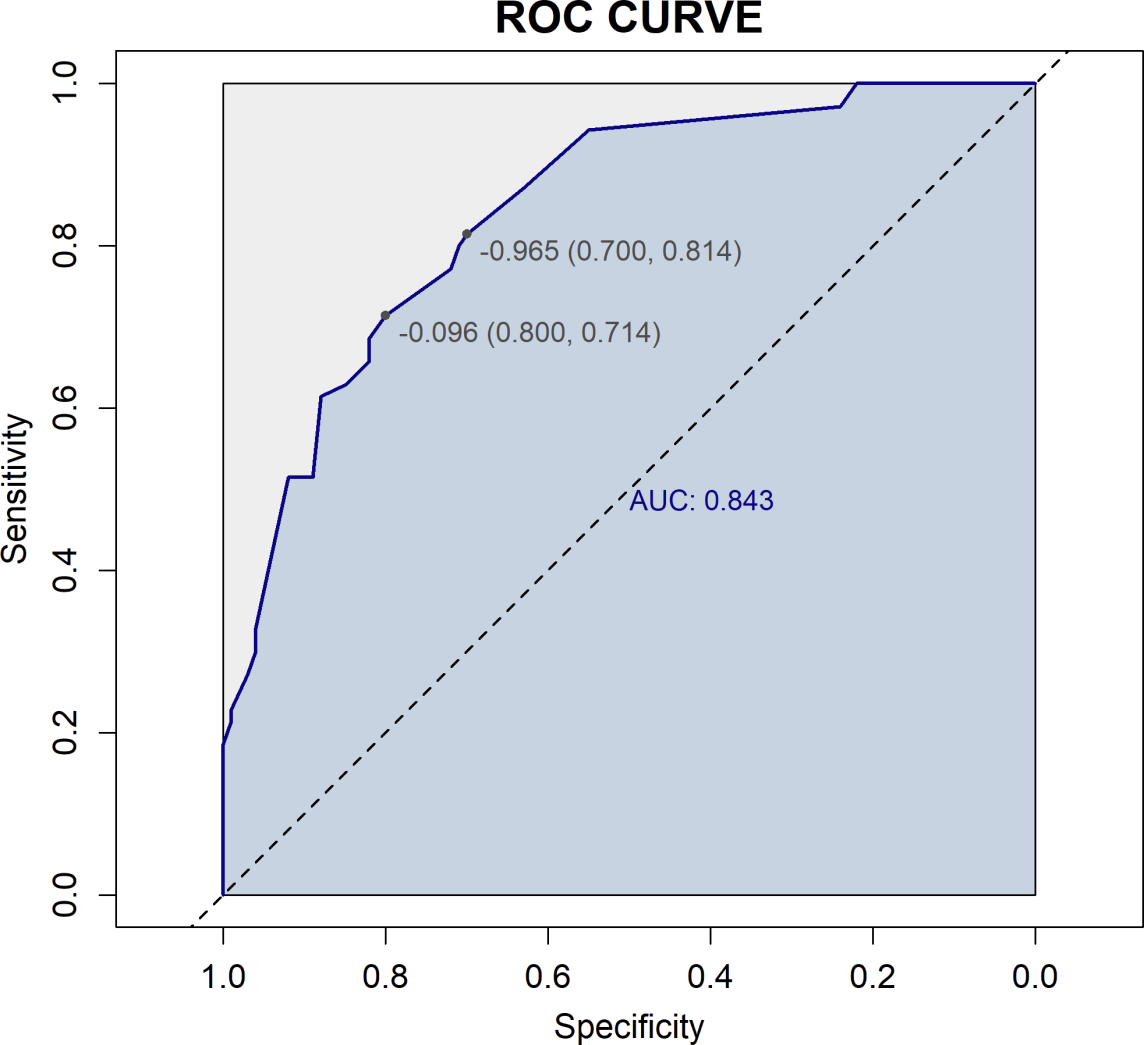
ROC curve of the prediction model. The horizontal axis (1-Specificity) represents the false positive rate, and the vertical axis (Sensitivity) represents the true positive rate. The dashed diagonal line represents random chance (AUC = 0.5). The blue curve is the ROC curve, which plots sensitivity against (1-specificity) at different threshold values. The AUC is 0.843, indicating good discriminative ability of the model in predicting 2-year recurrence risk.

**Figure 8:**
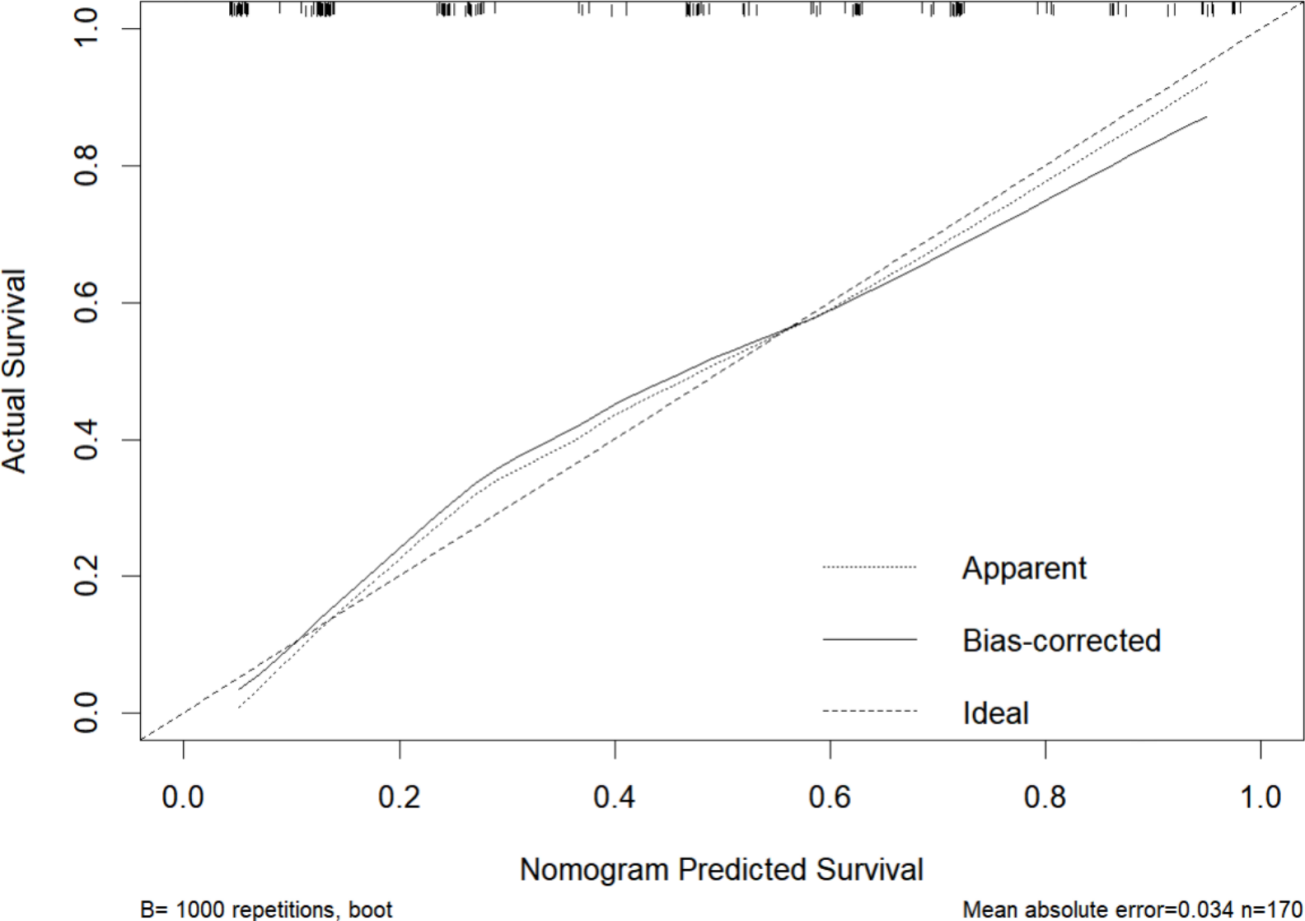
Calibration curve of the nomogram for 2-year recurrence risk prediction. The horizontal axis represents the nomogram-predicted survival probability for 2 years, and the vertical axis represents the actual survival probability based on observed outcomes. The dotted line (Apparent) represents the calibration curve based on the model’s performance on the training dataset, showing the apparent fit. The solid line (Bias-corrected) indicates the model’s calibration performance after adjusting for overfitting using bootstrapping (B=1000). The dashed line (Ideal) serves as the reference, representing perfect calibration where predicted probabilities exactly match the observed probabilities.

Based on four variables—surgical procedure, FIGO stage, completion of chemotherapy, and TCM exposure—this study developed a nomogram risk prediction model for the 2-year recurrence of EOC (**Figure 6**). The AUC of the prediction model was 0.843 (**Figure 7**). Internal validation was performed using the bootstrap method (1000 resampling iterations), and the 95% confidence interval was calculated to be 0.774-0.898. The Hosmer-Lemeshow goodness-of-fit test showed that the prediction model developed using the training set performed well in the overall dataset (*P* = 0.639). The calibration curve indicated good calibration of the model, as shown in **Figure 8**.

### 3.4 Sensitivity analysis

**Figure 9:**
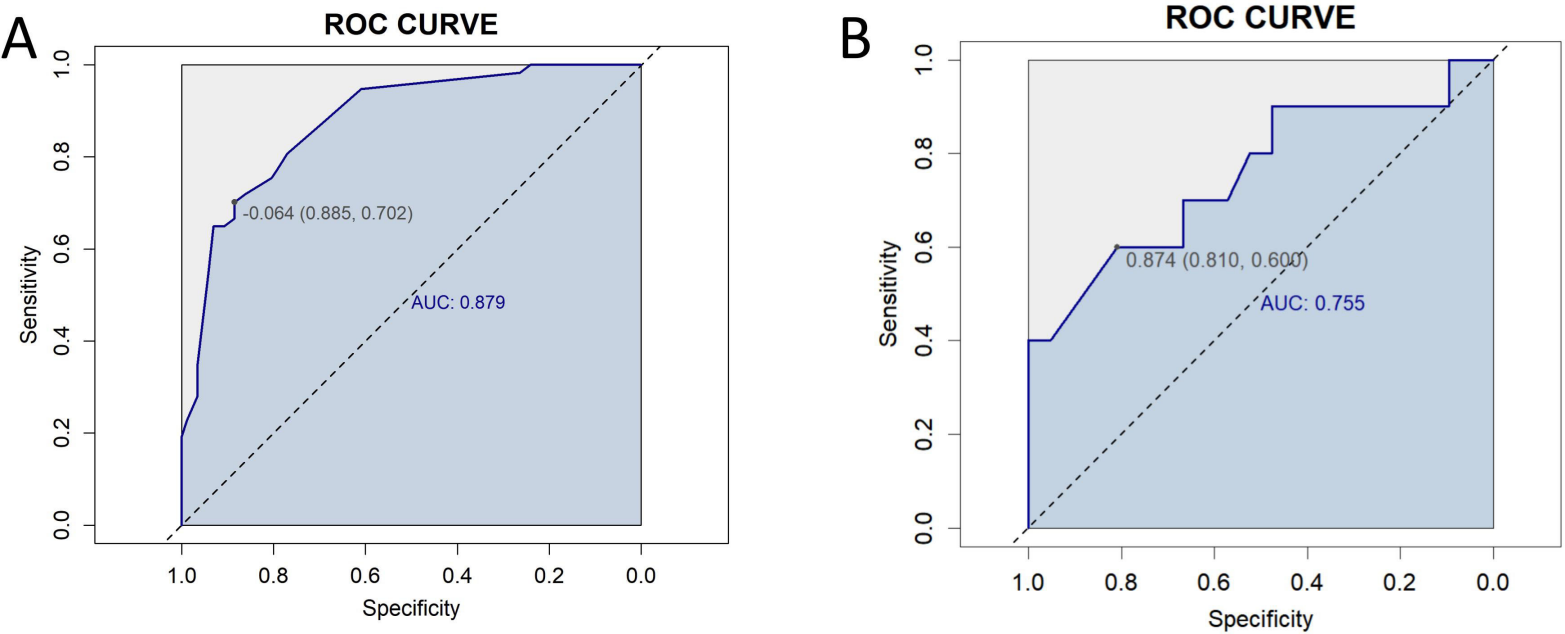
Receiver Operating Characteristic (ROC) curves for sensitivity analysis. **(A)** ROC curve for the complete-case dataset (n = 144), showing an area under the curve (AUC) of 0.879 (95% CI: 0.821–0.932). **(B)** ROC curve for the dataset including BRCA/HRD genetic testing data (n = 31), with an AUC of 0.755 (95% CI: 0.456–0.943).

We conducted sensitivity analyses to evaluate the discriminative performance of the model in two separate datasets: the complete-case dataset (i.e., excluding all records with missing data, n = 144) and the dataset containing BRCA/HRD genetic testing information (n = 31).Receiver Operating Characteristic (ROC) curves were generated for both cohorts (**Figure 9**). The model achieved an AUC of 0.879 (95% CI: 0.821–0.932) in the complete-case dataset and 0.755 (95% CI: 0.456–0.943) in the genetic testing dataset. These results suggest that the prediction model maintained high discriminative ability in the complete-case dataset, while its performance showed a modest decline in the BRCA/HRD-tested subgroup.

### 3.5 Clinical benefit analysis

**Figure 10:**
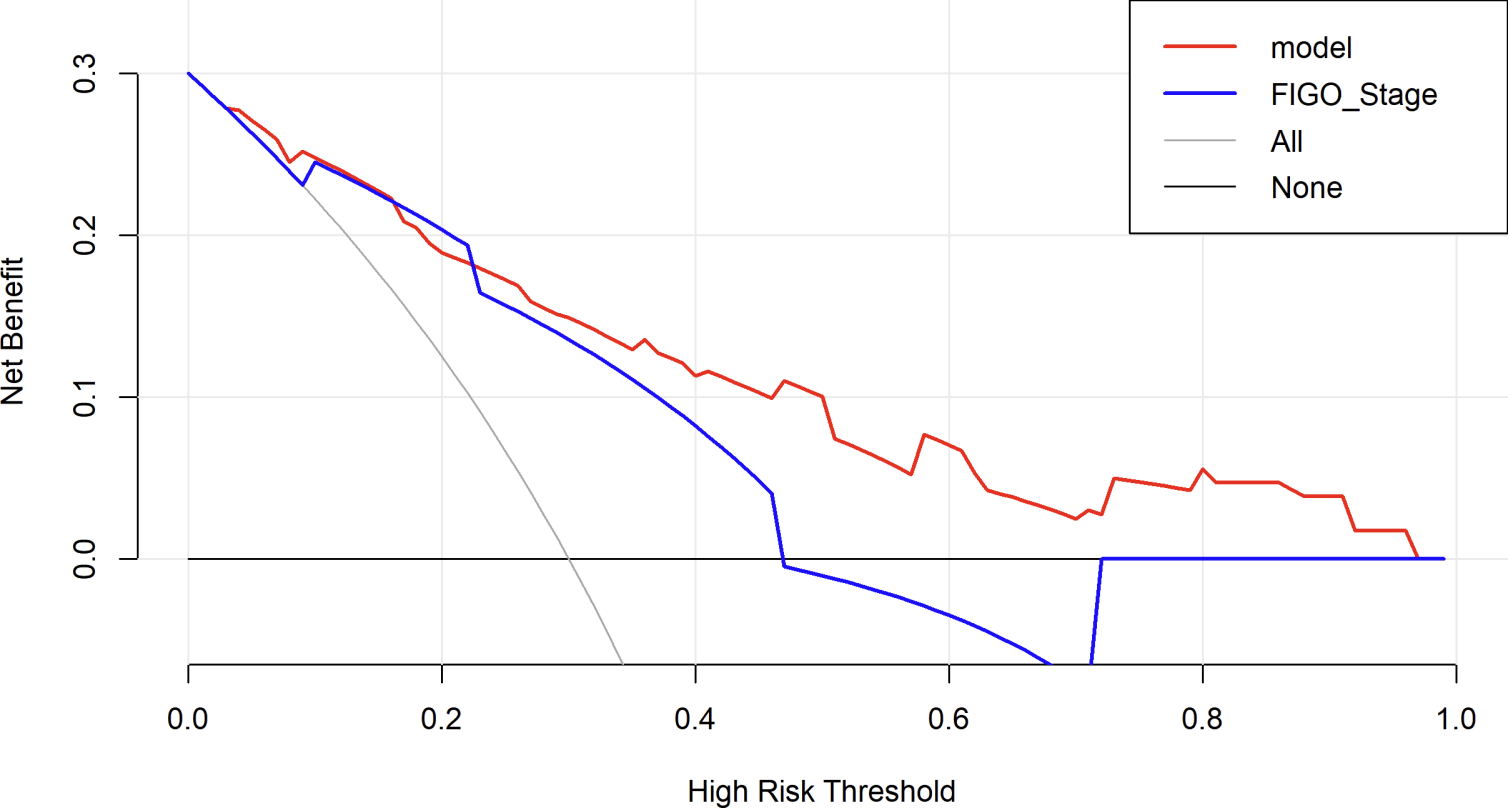
Decision curve analysis to assess the clinical utility of the proposed nomogram. The red line represents the net benefit of the nomogram model, while the blue line represents the FIGO staging system as a comparator. The black line (None) assumes no patients are treated, and the gray line (All) assumes all patients are treated. The decision curve shows that the nomogram provides a higher net benefit than FIGO staging and the “treat-all” or “treat-none” strategies across a clinically relevant range of risk thresholds.

DCA was performed to evaluate the clinical significance of the prediction model. The DCA of the EOC recurrence risk prediction nomogram is shown in **Figure 10**. The results indicate that the proposed model (red line) provides a higher net benefit across a wide range of high-risk threshold probabilities compared to the FIGO staging system (blue line), suggesting that its application in clinical decision-making may lead to better risk stratification and improved patient management.

## 4 Discussion

Ovarian cancer, as the leading cause of gynecological cancer-related mortality, is characterized by a high recurrence rate and mortality, making early prediction of patient prognosis a key focus in clinical practice. Several predictive models (16, 22) have been constructed using public databases. While these models provide general predictive tools, public data often lack many critical clinical variables, limiting the ability for personalized prognostic assessments. In contrast, this study utilizes case data from our center between 2011 and 2022, enabling a comprehensive collection of detailed clinical information, including detailed surgical records and chemotherapy records, to construct a more accurate recurrence risk prediction model. Furthermore, we innovatively incorporated TCM exposure as one of the influencing factors in the model, aiming to explore whether TCM intervention has a potential impact on patient prognosis. Our findings suggest that high exposure to TCM intervention significantly influences recurrence prediction, providing valuable insights into the potential role of integrated TCM and conventional medicine in the treatment of advanced ovarian cancer.

In this study, univariate ROC analysis was performed for all potential predictors, and the AUC values were visualized. The results showed that, except for the FIGO stage (AUC=0.760), none of the individual variables demonstrated significant predictive value for recurrence within 2 years in patients with EOC (0.25 < AUC < 0.75). However, relying solely on the FIGO stage for prediction was insufficient to accurately reflect the recurrence risk. Therefore, we further incorporated more potential prognostic factors and selected variables with p < 0.1 through univariate logistic regression. These variables were then included in LASSO regression to eliminate multicollinearity and simplify the model. Multivariate logistic regression identified four core variables: surgical procedure, FIGO stage, completion of chemotherapy, and TCM exposure. These variables are easily obtainable in clinical practice, making the model more convenient and practical. The four-variable model increased the AUC from 0.760 to 0.843, significantly improving the model’s discriminative ability. In previous studies, Qiaqia Li et al. (15) and Jun Hu et al. (16) also developed predictive models for recurrence in patients with EOC, with FIGO stage being one of the core variables in the model construction. However, this study introduced the LASSO regression machine learning algorithm, which effectively handles multicollinearity and simplifies the model, resulting in fewer and more representative predictive variables. For example, in the studies by Qiaqia Li et al. and Jun Hu et al., organ metastasis or lymph node metastasis were included as core predictive variables. However, due to the correlation between these variables and FIGO stage, their representativeness was somewhat diminished. In contrast, the four core variables selected in this study are simpler and exhibit higher independence, thereby enhancing the overall predictive capability of the model.

The nomogram, widely used as a tool for cancer prognosis prediction, has the advantage of visually quantifying multiple clinical variables, making the prediction results more personalized and clinically applicable. Compared to traditional staging systems (such as FIGO staging system or the American Joint Committee on Cancer Cancer Staging System), the nomogram integrates specific information from each patient into a total score, enabling a more precise individualized prognostic assessment (23). In this study, by combining four variables—surgical procedure, FIGO stage, chemotherapy completion, and TCM exposure—we developed a nomogram model to predict the recurrence risk of EOC, aiming to provide a more reliable prediction for recurrence risk within 2 years. The model demonstrated high discrimination and calibration, and DCA showed that the nomogram provided consistently higher net benefits compared to the FIGO staging system across a range of high-risk threshold probabilities. This suggests that the proposed model offers superior clinical utility in identifying high-risk patients and may aid in developing personalized follow-up and treatment strategies.

TCM exerts its therapeutic effects through multi-target, multi-component, and multi-pathway mechanisms, playing an important role in suppressing tumor recurrence and metastasis. Preclinical studies have demonstrated that TCM and its active constituents can inhibit the proliferation of ovarian cancer cells, promote apoptosis, reverse drug resistance, and modulate key oncogenic signaling pathways, including PI3K/Akt/mTOR, Wnt/β-catenin, Notch, NF-κB, and MAPK pathways (24–28). Building on these mechanistic insights, clinical studies have further supported the adjuvant role of TCM in ovarian cancer treatment, showing benefits such as improved quality of life and prolonged overall survival (OS). For instance, a randomized controlled trial by Yifan Li et al. (29) demonstrated that Yiqi Huoxue Jiedu Decoction not only improved the quality of life of patients with stage IIIc EOC, but also prolonged PFS. Moreover, a study by Qinglin Zhang et al. (12) found that the death risk of advanced ovarian cancer patients was significantly reduced after receiving TCM-based treatment. These studies provide evidence supporting the use of TCM in ovarian cancer treatment. However, most previous research has focused on improving quality of life and extending OS, and there has been limited investigation into the effect of TCM intervention on ovarian cancer recurrence. Therefore, this study is the first to incorporate TCM exposure as an intervention factor into a recurrence prediction model for ovarian cancer and to validate its unique impact on PFS through multivariate regression analysis. The results of this study show that TCM exposure significantly predicts recurrence risk, particularly the association between low TCM exposure and higher recurrence risk (OR=2.790, 95%CI=1.20-6.52, *P*=0.018). This suggests that high TCM exposure may play a potential role in prolonging PFS and reducing recurrence risk. The innovative aspect of this study lies in filling the research gap in the use of TCM for ovarian cancer recurrence prevention and treatment, providing new perspectives and directions for future research. It also further supports the potential application of TCM in cancer recurrence prevention, warranting deeper exploration and validation in the future.”

Although the nomogram prediction model developed in this study demonstrates good performance in predicting the recurrence risk of EOC, there are several limitations. ① As this study is based on retrospective medical records, the classification of TCM exposure may be subject to misclassification bias. ② The sample size of this study is relatively small, and the data were derived from a single center, lacking external validation from multiple centers. This may limit the generalizability of the model, restricting its applicability to other regions or different patient populations. ③ This study only performed basic binary classification of TCM exposure, without exploring the impact of different dosages and treatment durations on recurrence risk. ④ Previous studies have shown that BRCA mutations and HRD status are closely related to the sensitivity of ovarian cancer patients to PARP inhibitors and recurrence risk (30). BRCA mutations, particularly common in serous ovarian cancer, may indicate better treatment response and prolonged PFS (31). In our study, due to the limited availability of BRCA and HRD testing in mainland China, only a small subset of patients underwent genetic testing. While these indicators were not included in the main prognostic model, we performed a sensitivity analysis in this subgroup, which showed a modest decline in model discrimination. This finding highlights the potential prognostic value of genetic testing and suggests that incorporating BRCA/HRD status may further improve the predictive performance of the model. Future multicenter studies with broader genetic data collection are warranted to validate and enhance our findings. ⑤ The samples in this study span approximately 10 years, during which disease management, including surgical approaches and chemotherapy regimens, has changed, which may have influenced the prognosis. ⑥ Different TCM practitioners may have varying treatment approaches, and prescriptions are adjusted based on the patient’s real-time condition, leading to substantial heterogeneity. This study aims to provide an initial exploration of PFS in patients exposed to TCM, but specific prescriptions and dosages still require further prospective trials for validation.

To improve the generalizability and predictive accuracy of the model, future studies should conduct external validation of this model with multi-center, large-sample research. Further refinement of TCM exposure grouping, along with the inclusion of additional clinical and molecular biological data—particularly genetic and molecular biomarkers—will be necessary to optimize the structure and performance of the prediction model. This will, in turn, promote its practical application in the individualized treatment and recurrence prevention of ovarian cancer patients.

## 5 Conclusion

In this study, we developed a nomogram model for predicting the 2-year recurrence risk in patients with EOC. The model demonstrated good discrimination and calibration. Additionally, our findings provide preliminary evidence suggesting that long-term TCM exposure (≥6 months) may help extend PFS, although further research is needed to confirm this effect.

## Data Availability

The original contributions presented in the study are included in the article/supplementary material. Further inquiries can be directed to the corresponding author.
